# Sexually transmitted coinfections among at-risk HIV-positive MSM: implications for optimal preemptive treatment

**DOI:** 10.3389/fmed.2024.1328589

**Published:** 2024-03-15

**Authors:** Tzong-Yow Wu, Kuan-Yin Lin, Li-Hsin Su, Hsin-Yun Sun, Yu-Shan Huang, Wang-Da Liu, Wen-Chun Liu, Lan-Hsin Chang, Sui-Yuan Chang, Chien-Ching Hung

**Affiliations:** ^1^Department of Internal Medicine, National Taiwan University Hospital and National Taiwan University College of Medicine, Taipei, Taiwan; ^2^Department of Internal Medicine, National Taiwan University Hospital Yunlin Branch, Yunlin, Taiwan; ^3^Department of Medicine, National Taiwan University Cancer Center, Taipei, Taiwan; ^4^Department of Laboratory Medicine, National Taiwan University Hospital and National Taiwan University College of Medicine, Taipei, Taiwan; ^5^Department of Clinical Laboratory Sciences and Medical Biotechnology, National Taiwan University College of Medicine, Taipei, Taiwan; ^6^Department of Tropical Medicine and Parasitology, National Taiwan University College of Medicine, Taipei, Taiwan

**Keywords:** sexually transmitted coinfection, syphilis, *Chlamydia trachomatis*, *Neisseria gonorrhoeae*, *Mycoplasma*, *Ureaplasma*, polymerase chain reaction

## Abstract

**Background:**

Concurrent sexually transmitted infections (STIs) are common in sexually active populations. We aimed to estimate the prevalence and coinfection rates of bacterial STIs among sexually active, HIV-positive men who have sex with men (MSM), and to assess the potential benefits of different combination treatment regimens in managing concurrent bacterial STIs.

**Methods:**

From September 2021 to September 2023, HIV-positive MSM underwent STI testing when they had symptoms suggestive of STIs or recently acquired hepatitis C virus (HCV) infection or early syphilis. The oral rinse, rectal swab, and urethral swab specimens were tested for *Chlamydia trachomatis*, *Neisseria gonorrhoeae*, *Mycoplasma* spp., *Ureaplasma* spp., and *Trichomonas vaginalis* with the use of multiplex real-time polymerase-chain-reaction assays. The estimated coinfection rates were used to evaluate the benefits of different combination treatment regimens for managing coinfections.

**Results:**

During the study period, 535 participants (median age, 37 years; and CD4 count, 615 cells/mm^3^) were enrolled. On their first visits, at least one bacterial pathogen was detected in 57.9% and concomitant bacterial infections were found in 32.9% of the participants. The most commonly identified pathogen was *U. urealyticum* (36.3%), followed by *C. trachomatis* (22.8%), and *N. gonorrhoeae* (19.8%). The factors associated with any bacterial STIs included older age (per 1-year increase, adjusted odds ratio [AOR], 0.97; 95% confidence interval [CI], 0.95–1.00), early syphilis (AOR, 1.87; 95% CI, 1.22–2.84), and having more than 5 sex partners in the preceding 3 months (AOR, 2.08, 95% CI, 1.07–4.06). A combination therapy of benzathine penicillin G with a 7-day course of doxycycline could simultaneously treat 27.1% of *C. trachomatis* coinfections in participants with early syphilis, while a combination therapy of ceftriaxone with doxycycline could simultaneously treat 40.6% of chlamydial coinfections in participants with gonorrhea.

**Conclusion:**

Bacterial STIs were prevalent and concomitant infections were not uncommon among sexually active, HIV-positive MSM, supporting regular screening for bacterial STIs. The effectiveness of preemptive use of doxycycline as combination therapy for concurrent STIs warrants more investigations.

## Introduction

Sexually transmitted infections (STIs) have been on the increase over the past 20 years, which may be related to the adoption of biomedical HIV interventions and changes in sexual behaviors (1). Of bacterial STIs, *Treponema pallidum*, *Chlamydia trachomatis* and *Neisseria gonorrhoeae* are the most prevalent pathogens among men who have sex with men (MSM) (2–4). Several cell wall-less members of the Mollicutes class, namely *Mycoplasma genitalium*, *M. hominis*, *Ureaplasma urealyticum,* and *U. parvum*, are also associated with urogenital infections. While *M. genitalium* has been demonstrated to cause urethritis, proctitis, and epididymitis (5) and *U. urealyticum* may cause urethritis (6), *M. hominis* and *U. urealyticum* are thought to be related to male infertility (7).

Bacterial sexually transmitted coinfections are not uncommon in at-risk populations. It has been shown that 20–42% of individuals with *N. gonorrhoeae* infections have *C. trachomatis* coinfections (8). Among sexually active, HIV-positive MSM, we previously have found high rates of *C. trachomatis* and/or *N. gonorrhoeae* coinfections in those with recent hepatitis C virus (HCV) infection (50%) and incident syphilis (36%) (4). A sub-study nested in a randomized controlled trial evaluating the treatment response of early syphilis also demonstrated a high rate of sexually transmitted coinfections (27%) in patients with early syphilis, including chlamydial infection, gonorrhea, and non-gonococcal urethritis (9). With the advances in polymerase-chain-reaction (PCR) technology, multiplex PCR assay can amplify several different DNA sequences simultaneously and enable rapid detection of sexually transmitted coinfections. Furthermore, multi-site testing of specimens from oropharyngeal, urethral, rectal and vaginal sites has shown to facilitate the detection of STIs in both symptomatic and asymptomatic individuals (10, 11).

Although current STI guidelines recommend quarterly screening for chlamydia, gonorrhea, and syphilis for sexually active MSM to improve case finding and reduce onward transmission, STI screening in this population has been reported to be suboptimal (12, 13). While symptomatic STIs prompt immediate treatment, asymptomatic concurrent STIs may remain untreated due to insufficient screening and limitations of the cost incurred and the time required for PCR test results to be available. Therefore, adding doxycycline alongside ceftriaxone is suggested in the management of gonorrhea, especially when there is a significant risk of coinfection with chlamydial infection (12). However, the appropriate preemptive treatments for other concurrent STIs are not well defined. This study aimed to estimate the prevalence and coinfection rates of bacterial STIs among sexually active, HIV-positive MSM in Taiwan, and to evaluate the potential benefit of different treatment combination regimens in managing concurrent bacterial STIs.

## Methods

### Study population and setting

This prospective study was conducted at the National Taiwan University Hospital, a tertiary center for HIV care in northern Taiwan. During 2019–2020, we implemented a screening program for *N. gonorrhoeae* and *C. trachomatis* infections among sexually active people with HIV (PWH), which revealed high coinfection rates in those with recent HCV infection and syphilis (4). Therefore, the screening program was continued among at-risk PWH. From September 2021 to September 2023, we consecutively enrolled HIV-positive MSM aged 20 years or older who presented with symptoms suggestive of STIs, or recently acquired HCV infection within 1 year or early syphilis. The diagnoses of STIs, HCV infection, and syphilis were made based on clinical signs and confirmed through laboratory testing. According to the national guidelines for HIV treatment (13), PWH received serologic tests for syphilis, hepatitis B, and hepatitis C at the first visit for HIV care; afterwards, they received regular serologic tests for syphilis and viral hepatitis every 3–6 months and annually, respectively. The study was approved by the Research Ethics Committee of National Taiwan University Hospital (registration number, NTUH-201811021RINA), and all participants provided written informed consent for both STI testing and data collection prior to enrollment.

### Study procedures

The participants were provided with clear and specific instructions for proper self-collection of specimens, including oral rinse, rectal swabs, and urethral swabs. This process involved swishing and gargling with 10 mL of sterile water for 15 s for oral rinse samples, inserting a blind anal swab 3–5 cm into the anal canal and rotating it, and inserting a urethral swab 1 cm into the urethra and rotating it. The specimens were tested for 7 sexually transmitted pathogens, including *C. trachomatis*, *N. gonorrhoeae*, *M. genitalium*, *M. hominis*, *U. urealyticum*, *U. parvum*, and *Trichomonas vaginalis*. Bacterial STIs were diagnosed when specimens collected from any anatomical site were tested positive for any of these 7 pathogens by PCR assays. According to the STI treatment guidelines, participants who were diagnosed with STIs based on symptoms and those who were asymptomatic but tested positive for *C. trachomatis* or *N. gonorrhoeae* received antibiotic treatment (12). While screening and treatment of asymptomatic *Mycoplasma* and *Ureaplasma* infection is not recommended by current STI guidelines (12, 14), the decision to treat individuals who tested positive for *Mycoplasma* and *Ureaplasma* spp. was at the discretion of the treating physicians. Because diagnostic information might not be immediately available, a preemptive 7-day course of doxycycline was recommended for participants with urethritis and proctitis based on potential pathogens and for those with gonorrhea due to the high coinfection rate with chlamydia (12). Participants with concurrent STIs and not receiving preemptive antibiotic treatment might require additional follow-ups as outpatients to receive the appropriate treatment. Participants underwent initial screening for STIs before receiving either preemptive or definitive treatment. A test of cure to detect treatment failure was not regularly performed unless adherence to treatment administered was uncertain or symptoms persisted (12). Some participants were diagnosed with HCV infection at enrolment through their participation in another study for early detection of acute HCV infection with the use of pooled-plasma HCV RNA testing (15).

The participants were asked to complete a self-administered questionnaire interview, which assessed their socioeconomic status, symptoms suggestive of STIs (genital or anal lesions, dysuria, urogenital or rectal discharge, and diarrhea with pus, mucus or blood), previous history of STIs within 1 year, sexual behaviors in the preceding 3 months (such as the number of sexual partners, sexual preference, sexual activity, frequency of condom use, chemsex, internet-based sex, and transactional sex), and substance use in the preceding year (such as the use of 3,4-methylenedioxy-N-methylamphetamine, rush poppers, ketamine, marijuana, amphetamine, cocaine, and heroin).

### Laboratory investigations

The oral rinse and urethral and rectal swab specimens were collected using the specimen collection kit and tested with the use of multiplex real-time PCR assay (Allplex™ STI Essential Assay; Seegene Inc., Seoul, Republic of Korea) to identify *C. trachomatis*, *N. gonorrhoeae*, *M. genitalium*, *M. hominis*, *U. urealyticum*, *U. parvum* and *T. vaginalis*. The Seegene assay has an overall sensitivity of 98% and specificity of 94% (16, 17). High concordance of results was shown in the testing of urine samples, urethral swabs, genital swabs, and semen samples between the assay and agar culture, PCR amplification, and next-generation sequencing (18). CD4 cell count was determined using flow cytometry (BD FACS Calibur; Becton Dickinson, CA, United States), while plasma HIV RNA load (PVL) was quantified using Cobas AmpliPrep/Cobas TaqMan HIV-1 test (version 2.0; Roche Molecular Systems) with a lower detection limit of 20 copies/ml. The diagnosis of syphilis involved clinical evaluation of symptoms and serologic tests, including a reactive rapid plasma reagin test (BD Macro-VueTMRPR Card tests) for screening and confirmation using the *Treponema pallidum* particle agglutination assay (FTI-SERODIA-TPPA; Fujirebio Taiwan Inc., Taoyuan, Taiwan). Hepatitis B virus surface antigen (HBsAg) and HCV antibody were measured with the use of an enzyme immunoassay (Abbott Laboratories, Abbott Park, IL). To detect acute HCV infection and reinfection, plasma HCV RNA was determined using Cobas AmpliPreP HCV test (version 2.0; Roche Molecular Systems, Pleasanton, CA, United States) (15).

### Statistical analysis

Categorical variables were compared using the chi-squared test or Fisher’s exact test, whereas continuous variables were analyzed using a Mann–Whitney U test. A logistic regression model was conducted to identify factors associated with bacterial STIs. For multivariable analyzes, we included variables with a *p* value <0.1 in univariable analyzes, and then used a backward stepwise selection to determine the final model. The effects of each variable on acquisition of bacterial STIs were estimated with 95% confidence intervals (CIs) of odds ratios (ORs). A two-tailed *p* value <0.05 was considered statistically significant. All statistical analyzes were performed using Stata 17.0 (Stata Corporation, College Station, TX, United States).

## Results

During the 2-year study period, a total of 535 HIV-positive MSM were enrolled. The overall rate of participants with at least one bacterial STI pathogen detected at any anatomical site was 57.9% (310/535). Table 1 summarizes the baseline characteristics of the participants with and those without bacterial STIs. Overall, the participants had a median age of 37 years and a median CD4 count of 615 cells/mm^3^; and 94.4% of them had achieved PVL <200 copies/mL with antiretroviral therapy. At screening, 8.2% had symptoms of STIs; 8.2% tested positive for hepatitis B surface antigen and 25.0% tested positive for HCV antibody and/or RNA; and 59.3% received a diagnosis of early syphilis. Compared with participants without bacterial STIs, those with bacterial STIs were more likely to be younger (median age, 36 vs. 38 years, *p* = 0.004) and to have a history of syphilis (88.1% vs. 81.8%, *p* = 0.042) and early syphilis (64.5% vs. 52.0%, *p* = 0.004), symptoms suggestive of STIs (10.7% vs. 4.9%, *p* = 0.017), substance use (47.6% vs. 35.5%, *p* = 0.010), more than 5 sex partners in the past 3 months (17.6% vs. 9.0%, *p* = 0.009), and inconsistent condom use (75.6% vs. 63.4%, *p* = 0.005).

**Table 1:** Baseline characteristics of the participants.

	Total (*N* = 535)	Bacterial STIs (*N* = 310)	No bacterial STIs (*N* = 225)	*p*-value
*Clinical characteristics*				
Age, median (IQR), years	37 (32–43)	36 (32–42)	38 (33–45)	0.004
HBsAg positivity, *n* (%)	44 (8.2)	29 (9.4)	15 (6.7)	0.264
HCV infection^a^, *n* (%)	134 (25.0)	83 (26.8)	51 (22.7)	0.279
History of syphilis, *n* (%)	457 (85.4)	273 (88.1)	184 (81.8)	0.042
Early syphilis^b^, *n* (%)	317 (59.3)	200 (64.5)	117 (52.0)	0.004
Late syphilis^c^, *n* (%)	140 (26.2)	73 (23.5)	67 (29.8)	0.106
Receiving cART at screening, *n* (%)	521 (97.4)	301 (97.1)	220 (97.8)	0.626
CD4 count at screening, median (IQR), cells/mm^3^	615 (473–815)	615 (469–815)	615 (478–798)	0.836
PVL at screening, median (IQR), log_10_ copies/mL	UD^d^ (UD-UD)	UD^d^ (UD-UD)	UD^d^ (UD-UD)	0.228
PVL <200 copies/mL at screening, *n* (%)	505 (94.4)	290 (93.6)	215 (95.6)	0.319
*Questionnaire interview*				
Symptoms of STIs at screening, *n* (%)	44/535 (8.2)	33/310 (10.7)	11/225 (4.9)	0.017
Previous STIs within 1 year, *n* (%)	299/535 (55.9)	180/310 (58.1)	119/225 (52.9)	0.234
University or higher education, *n* (%)	392/464 (84.5)	226/274 (82.5)	166/190 (87.4)	0.153
Full-time employment, *n* (%)	381/462 (82.5)	227/273 (83.2)	154/189 (81.5)	0.643
Monthly income >1,600 USD, *n* (%)	163/463 (35.2)	101/274 (36.9)	62/189 (32.8)	0.369
Substance use, *n* (%)	195/458 (42.6)	128/269 (47.6)	67/189 (35.5)	0.010
Anal-penile sex, *n* (%)	364/459 (79.3)	222/270 (82.2)	142/189 (75.1)	0.065
Oral-penile sex, *n* (%)	338/459 (73.6)	207/270 (76.7)	131/189 (69.3)	0.078
Oral-anal sex, *n* (%)	178/451 (39.5)	108/267 (40.5)	70/184 (38.0)	0.607
Partner known to have STI, *n* (%)	220/442 (49.8)	139/261 (53.3)	81/181 (44.8)	0.079
Number of sex partners >5 within 3 months, *n* (%)	65/463 (14.0)	48/273 (17.6)	17/190 (9.0)	0.009
Inconsistent condom use, *n* (%)	317/449 (70.6)	201/266 (75.6)	116/183 (63.4)	0.005
Use of mobile dating application, *n* (%)	267/450 (59.3)	166/269 (61.8)	101/181 (55.8)	0.211
Chemsex, *n* (%)	81/455 (17.8)	56/272 (20.6)	25/183 (13.7)	0.058

CART, combination antiretroviral therapy; HbsAg, hepatitis B surface antigen; HCV hepatitis C virus, IQR, interquartile range; PVL, plasma HIV RNA load; STI, sexually transmitted infection; UD, undetectable; USD, United States Dollar.

a Participants testing positive for anti-HCV IgG and/or HCV RNA in the past 12 months.

b Early syphilis included primary, secondary and early latent syphilis. Participants presenting with ulcers in the oropharyngeal, anal, and genital regions were diagnosed with primary syphilis, while those with skin rashes, mucocutaneous lesions, or generalized lymphadenopathy were diagnosed with secondary syphilis. Early latent syphilis was defined as either seroconversion within the past 1 year; or, in cases of patients with a prior history of syphilis, a fourfold or higher increase in RPR titer, with the absence of syphilis symptoms.

c Late syphilis included late latent syphilis and latent syphilis of unknown duration, defined by the presence of reactive syphilis serology, either with no prior syphilis diagnosis or with a fourfold or higher increase in RPR titer in cases with a prior syphilis diagnosis; additionally, there should be an absence of symptoms and no evidence of acquiring the disease within the past year.

d PVL <20 copies/ml.

**Figure 1:**
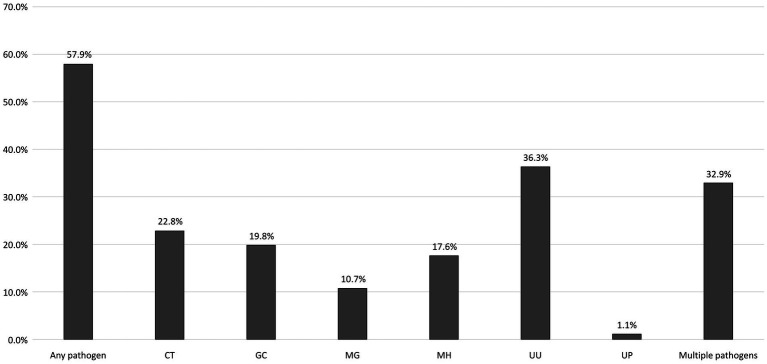
Proportions of bacterial STIs among the participants. STI, sexually transmitted infection; PLWH, people living with HIV; CT, *Chlamydia trachomatis*; GC, *Neisseria gonorrhoeae*; MG, *Mycoplasma genitalium*; MH, *Mycoplasma hominis*; UU, *Ureaplasma urealyticum*; UP, *Ureaplasma parvum*.

**Figure 2:**
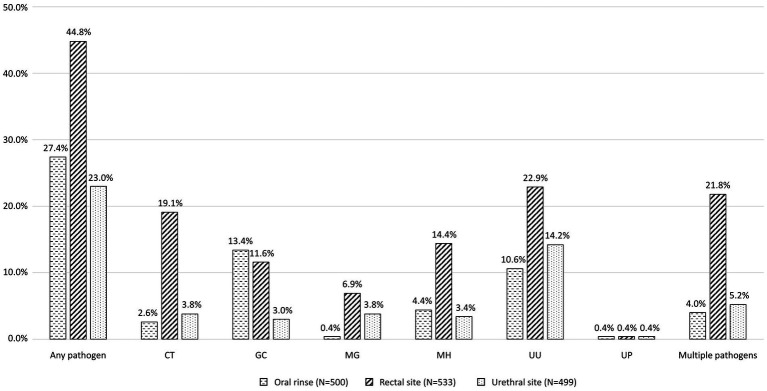
Proportions of bacterial STIs detected from different anatomical sites. STI, sexually transmitted infection; CT, *Chlamydia trachomatis*; GC, *Neisseria gonorrhoeae*; MG, *Mycoplasma genitalium*; MH, *Mycoplasma hominis*; UU, *Ureaplasma urealyticum*; UP, *Ureaplasma parvum.*

Figure 1 shows the proportions of different bacterial STIs identified from the participants. The most commonly identified pathogen was *U. urealyticum* (n = 194, 36.3%), followed by *C. trachomatis* (122, 22.8%), *N. gonorrhoeae* (106, 19.8%), *M. hominis* (94, 17.6%), *M. genitalium* (57, 10.7%), and *U. parvum* (6, 1.1%). No participants tested positive for *T. vaginalis*. Figure 2 demonstrates the proportions of bacterial STIs detected from different anatomical sites. Most bacterial STIs were detected from rectal sites (239/533, 44.8%), followed by oral rinse (137/500, 27.4%) and urethral sites (115/499, 23.0%). While the detection rates of *C. trachomatis*, *M. genitalium*, *M. hominis*, and *U. urealyticum* were highest in specimens from rectal sites (83.6% [102/122], 64.9% [37/57], 81.9% [77/94], 62.9% [122/194], respectively), the detection rate of *N. gonorrhoeae* was highest in specimens from oral rinse (67/106, 63.2%). Concomitant bacterial infections were found in 32.9% (176/535) of the participants, especially in specimens from rectal sites (116/176, 65.9%). Factors associated with bacterial STIs are listed in Table 2. In multivariable analysis, the independent factors associated with bacterial STIs among HIV-positive MSM included early syphilis (AOR, 1.87; 95% CI, 1.22–2.84) and having more than 5 sex partners (AOR, 2.08; 95% CI, 1.07–4.06), while older age showed marginal association (per 1-year increase, adjusted OR [AOR], 0.97, 95% CI, 0.95–1.00). Further information regarding the factors associated with specific bacterial STIs is listed in Supplementary Tables S1–S6. In multivariable analysis, chlamydia was associated with early syphilis (AOR, 1.66; 95% CI, 1.05–2.63); gonorrhea was related to age (per 1-year increase, AOR, 0.94; 95% CI, 0.91–0.98) and symptoms of STIs (AOR, 4.00; 95% CI, 1.96–8.21), while the CD4 count (per 10-cell/mm^3^ increase, AOR, 1.01, 95% CI, 1.00–1.02) showed marginal association.

**Table 2:** Factors associated with bacterial STIs among the participants.

	Univariable analysis		Multivariable analysis^b^	
	OR (95% CI)	*p*-value	AOR (95% CI)	*p*-value
Age, per 1-year increase	0.97 (0.95–1.00)	0.005	0.97 (0.95–1.00)	0.045
HBsAg positivity	1.44 (0.75–2.75)	0.275		
HCV infection^a^	1.26 (0.84–1.88)	0.261		
History of syphilis				
Early syphilis	1.69 (1.19–2.40)	0.003	1.87 (1.22–2.84)	0.004
Late syphilis	0.73 (0.49–1.08)	0.111		
Receiving cART at screening	0.76 (0.25–2.30)	0.627		
CD4 count at screening, per 10-cell/mm^3^ increase	1.00 (0.99–1.00)	0.497		
PVL at screening, per 1-log copies/mL increase	1.11 (0.90–1.37)	0.333		
Symptoms of STIs at screening	2.32 (1.14–4.69)	0.019	2.30 (0.99–5.34)	0.052
Previous STIs within 1 year	1.23 (0.87–1.74)	0.234		
University or higher education	0.71 (0.40–1.25)	0.235		
Full-time employment	1.12 (0.69–1.82)	0.643		
Monthly income >1,600 USD	1.20 (0.81–1.77)	0.369		
Substance use	1.65 (1.13–2.42)	0.010	1.30 (0.79–2.15)	0.307
Anal-penile sex	1.53 (0.97–2.41)	0.066	0.75 (0.42–1.36)	0.346
Oral-penile sex	1.45 (0.96–2.21)	0.079	1.26 (0.79–2.00)	0.338
Oral-anal sex	1.11 (0.75–1.63)	0.607		
Partner known to have STI	1.41 (0.96–2.06)	0.079	1.07 (0.70–1.66)	0.747
Number of sex partners >5 within 3 months	2.17 (1.21–3.91)	0.010	2.08 (1.07–4.06)	0.031
Inconsistent condom use	1.79 (1.18–2.69)	0.006	1.64 (0.99–2.74)	0.057
Use of mobile dating application	1.28 (0.87–1.87)	0.211		
Chemsex	1.64 (0.98–2.74)	0.060	1.15 (0.60–2.22)	0.666

AOR, adjusted odds ratio; cART, combination antiretroviral therapy; CI, confidence interval; HBsAg, hepatitis B surface antigen; HCV hepatitis C virus; PVL, plasma HIV RNA load; STI, sexually transmitted infection; USD, United States dollars.

a Participants testing positive for anti-HCV IgG and/or HCV RNA in the past 12 months.

b The ORs are the estimates of the effect of covariates on bacterial STIs, adjusted for age, early syphilis, symptoms of STIs, substance use, anal-penile sex, oral-penile sex, partner infected with STI, number of sex partners, inconsistent condom use, and chemsex using a logistic regression model.

The detailed information on the coinfection rates of bacterial STI among the participants is shown in Table 3. Participants with early syphilis were frequently coinfected with *U. urealyticum* (133/317, 42.0%), followed by *C. trachomatis* (86/317, 27.1%) and *N. gonorrhoeae* (64/317, 20.2%). Participants testing positive for *C. trachomatis* were frequently coinfected with *U. urealyticum* (65/122, 53.3%) and *N. gonorrhoeae* (43/122, 35.2%), and those testing positive for *N. gonorrhoeae* were frequently coinfected with *U. urealyticum* (62/106, 58.5%) and *C. trachomatis* (43/106, 40.6%). Participants who tested positive for any bacterial STI pathogen had a notably high rate of concurrent early syphilis, ranging from 60.4 to 70.5%. To further characterize concurrent bacterial STIs among the participants with syphilis, Supplementary Figure S1 depicts the rates of bacterial STIs among those with and without early syphilis. The prevalence of bacterial STIs among the participants with early syphilis was significantly higher than that among those without early syphilis (63.1% vs. 50.0%, *p* = 0.003).

**Table 3:** Coinfection rates of bacterial STIs among the participants.

Bacterial STI pathogen	Coinfection rates of bacterial STIs (%)						
	Early syphilis	CT	GC	MG	MH	UU	UP
Early syphilis (*n* = 317)	–	86/317 (27.1)	64/317 (20.2)	37/317 (11.7)	66/317 (20.8)	133/317 (42.0)	4/317 (1.3)
CT (*n* = 122)	86/122 (70.5)	–	43/122 (35.2)	10/122 (8.2)	36/122 (29.5)	65/122 (53.3)	1/122 (0.8)
GC (*n* = 106)	64/106 (60.4)	43/106 (40.6)	–	12/106 (11.3)	35/106 (33.0)	62/106 (58.5)	2/106 (1.9)
MG (*n* = 57)	37/57 (64.9)	10/57 (17.5)	12/57 (21.1)	–	16/57 (28.1)	27/57 (47.4)	0/57 (0.0)
MH (*n* = 94)	66/94 (70.2)	36/94 (38.3)	35/94 (37.2)	16/94 (17.0)	–	67/94 (71.3)	3/94 (3.2)
UU (*n* = 194)	133/194 (68.6)	65/194 (33.5)	62/194 (32.0)	27/194 (13.9)	67/194 (34.5)	–	3/194 (1.5)
UP (*n* = 6)	4/6 (66.7)	1/6 (16.7)	2/6 (33.3)	0/6 (0.0)	3/6 (50.0)	3/6 (50.0)	–

CT, *Chlamydia trachomatis*; GC, *Neisseria gonorrhoeae*; MG, *Mycoplasma genitalium*; MH, *Mycoplasma hominis*; STI, sexually transmitted infection; UU, *Ureaplasma urealyticum*; UP, *Ureaplasma parvum*.

The potential benefits of different combination treatment regimens, including single-dose BPG plus 7-day doxycycline or single-dose azithromycin, single-dose ceftriaxone plus 7-day doxycycline or single-dose azithromycin, and 14-day doxycycline, in managing concurrent bacterial STIs according to the coinfection rates are listed in Supplementary Figure S2. A combination therapy with 7-day doxycycline, including single-dose BPG plus 7-day doxycycline as well as single-dose ceftriaxone plus 7-day doxycycline, could simultaneously treat 27.1% of chlamydial coinfections among participants with early syphilis and 40.6% among those with gonorrhea, as recommended treatment regimens (Supplementary Figures S2B,E). A combination therapy with azithromycin was not deemed sufficient as the first-line treatment choice for managing concurrent STIs due to concerns of resistance and clinical efficacy (Supplementary Figures S2C,E).

## Discussion

In this study of STI screening among sexually active, HIV-positive MSM who presented with symptoms suggestive of STIs, or recently acquired HCV infection or early syphilis, we found a high prevalence of bacterial STIs (57.9%), including *C. trachomatis*, *N. gonorrhoeae*, and Mollicutes infections. Sexually transmitted bacteria were more frequently identified from the rectal sites except *N. gonorrhoeae* that was mostly detected from oral rinse. Sexually transmitted coinfections were not uncommon. While 32.9% of the participants had concomitant bacterial STIs, 63.1% of the participants with early syphilis was coinfected with bacterial STIs. The use of combination therapy with doxycycline may provide potential benefits in the management of concurrent bacterial STIs.

Previous studies conducted among PWH consistently showed that the prevalence of *C. trachomatis*, *N. gonorrhoeae*, and Mollicutes infections was 20 to 30%, which was associated with a history of viral hepatitis and genital warts (4, 19–21). Our study performed STI screening among PWH at risk for STIs with the use of multiplex PCR assay to detect pathogens from different anatomical sites; therefore, the prevalence of *C. trachomatis*, *N. gonorrhoeae*, and Mollicutes infections (57.9%) was higher compared with those of previous studies. The results showed that more than 50% of PWH with symptoms of STIs and recent acquisition of syphilis or HCV infection had bacterial STIs, suggesting the need for active STI surveillance in the at-risk populations.

*C. trachomatis, N. gonorrhoeae*, and *M. genitalium* are frequently reported to cause symptomatic urethritis and chronic complications among men (12). Previous studies showed that the prevalence of *C. trachomatis, N. gonorrhoeae*, and *M. genitalium* infections varied in different populations and settings. Of them, *C. trachomatis* infection has been considered as the most prevalent STI, with the estimated prevalence of 5–10% in PWH (2, 19, 20). In PWH with a history of STI or symptoms suggestive of an STI, the prevalence will increase to 11–25% (4, 21). Our study also demonstrated a similar prevalence of *C. trachomatis, N. gonorrhoeae*, and *M. genitalium* infections. Although the clinical significance of *Ureaplasma* species identified in our study has been debated, *U. urealyticum* was found to be an etiological agent of non-gonococcal urethritis in a meta-analysis (6) and the clinical significance of *U. parvum* in non-gonococcal urethritis was supported by a cohort study (22). While previous studies showed that the prevalence of *U. urealyticum* infection ranged from 9 to 22% in PWH (19–21), our study demonstrated that *U. urealyticum* was the predominant pathogen in PWH, with a higher prevalence compared with those reported in previous studies. However, most participants with *U. urealyticum* were asymptomatic in our study (178/194, 91.8%).

In consistent with previous studies, our study demonstrated a high percentage of bacterial coinfections among sexually active, HIV-positive MSM, especially those with early syphilis (4, 9). We previously found high rates of *C. trachomatis* and *N. gonorrhoeae* coinfections in PLWH with recent HCV infection (38 and 27%, respectively) and incident syphilis (29 and 16%, respectively) (4). Another study using data from a large US commercial laboratory showed that current or past syphilis was significantly more common among MSM testing positive for rectal gonorrhea or chlamydia than those testing negative (24% vs. 13%) (23). Furthermore, a previous study found that, in cases of newly diagnosed syphilis, MSM had a higher proportion of concurrent *C. trachomatis* or *N. gonorrhoeae* infection compared to their heterosexual counterparts (20% vs. 4%) (24). It is known that syphilis is re-emerging globally due to changes in sexual behaviors and the implementation of biomedical HIV interventions, particularly among MSM and other vulnerable populations (25–27). Given the high rate of STI coinfection among MSM with early syphilis, alongside regular syphilis screening being a crucial aspect of STI care (12), there is a need to integrate screening for concurrent bacterial STIs among MSM with early syphilis. This approach may enhance the early detection of concurrent STIs.

The findings that 20–42% of individuals infected with *N. gonorrhoeae* were coinfected with *C. trachomatis* have led to the recommendation that individuals treated for gonococcal infection should also receive anti-chlamydial therapy if chlamydial infection has not been excluded (12). The other rationale of the recommendation has also been attributed to the concerns about cost and turnaround time of microbiologic diagnostics. In addition, asymptomatic extragenital infections are prevalent among MSM; thus, diagnoses will be missed if pharyngeal and anorectal screenings are not performed (28–31). Although sampling from multiple anatomical sites could yield a higher detection rate, the testing cost incurred also increases (32). Considering the high rates of chlamydial coinfection among sexually active, HIV-positive MSM who acquired early syphilis, a preemptive 7-day course of doxycycline in combination with single-dose BPG may offer prompt treatment for chlamydial coinfection, minimize the need for additional follow-up visits and expenses, and reduce the risk of onward transmission. Furthermore, a recent study among patients with early syphilis revealed that treatment with single-dose BPG plus 7-day doxycycline could achieve a higher serologic response rate in syphilis treatment compared to treatment with BPG alone (33). Nevertheless, there is a theoretical risk that increased use of doxycycline could lead to the development of drug resistance and affect the microbiota, including *Staphylococcus aureus*, commensal *Neisseria* species, and other organisms within the gut flora (34). Although resistance to doxycycline is exceptionally rare for *C. trachomatis* and *T. pallidum*, there have been reports of antimicrobial resistance in *M. genitalium* (35), warranting more investigations into the adverse impact of wider use of doxycycline.

There are several limitations of the current study. First, missing data and recall biases in the questionnaire interview might mislead the evaluation of associations between bacterial STIs and socioeconomic status and sexual behaviors in this study. Second, the information on insertive and receptive roles during sexual intercourse were not collected, which precluded us from analyzing the association with oral transmission. Third, we did not evaluate the cost-effectiveness and the potential impact on resistance related to the use of pre-emptive doxycycline in combination therapy. However, it’s notable that the expense associated with the multiplex real-time PCR assay significantly exceeded that of a 7-day course of doxycycline. Finally, the included participants were sexually active, HIV-positive MSM and therefore, our findings may not be generalizable to other populations.

In conclusion, bacterial STIs were prevalent and concomitant infections were not uncommon in multiple anatomic sites among sexually active, HIV-positive MSM. Our findings highlight the importance of regular screening of bacterial STIs using PCR assays and combination treatment regimens for concurrent bacterial STIs, which could contribute to reduced risk of onward transmission.

## Data availability statement

The raw data supporting the conclusions of this article will be made available by the authors, without undue reservation.

## Ethics statement

The studies involving humans were approved by Research Ethics Committee of National Taiwan University Hospital (registration number, NTUH-201811021RINA). The studies were conducted in accordance with the local legislation and institutional requirements. The participants provided their written informed consent to participate in this study.

## Author contributions

T-YW: Writing – original draft. K-YL: Writing – original draft. L-HS: Writing – original draft. H-YS: Writing – original draft. Y-SH: Writing – original draft. W-DL: Writing – original draft. W-CL: Writing – original draft. L-HC: Writing – original draft. S-YC: Writing – original draft. C-CH: Writing – review & editing.

## References

[1] Chow EPF Grulich AE Fairley CK. Epidemiology and prevention of sexually transmitted infections in men who have sex with men at risk of HIV. Lancet HIV. (2019) 6:e396–405. doi: 10.1016/S2352-3018(19)30043-8 31006612

[2] Jansen K Steffen G Potthoff A Schuppe AK Beer D Jessen H. Group MSMSS. STI in times of PrEP: high prevalence of chlamydia, gonorrhea, and mycoplasma at different anatomic sites in men who have sex with men in Germany. BMC Infect Dis. (2020) 20:110. doi: 10.1186/s12879-020-4831-4, PMID: 32033533 PMC7007644

[3] Read TRH Murray GL Danielewski JA Fairley CK Doyle M Worthington K. Symptoms, sites, and significance of *Mycoplasma genitalium* in men who have sex with men. Emerg Infect Dis. (2019) 25:719–27. doi: 10.3201/eid2504.181258, PMID: 30882306 PMC6433010

[4] Lin KY Sun HY Lee TF Chuang YC Wu UI Liu WC. High prevalence of sexually transmitted coinfections among at-risk people living with HIV. J Formos Med Assoc. (2021) 120:1876–83. doi: 10.1016/j.jfma.2020.12.008, PMID: 33341349

[5] Bachmann LH Kirkcaldy RD Geisler WM Wiesenfeld HC Manhart LE Taylor SN. Prevalence of *Mycoplasma genitalium* infection, antimicrobial resistance mutations, and symptom resolution following treatment of urethritis. Clin Infect Dis. (2020) 71:e624–32. doi: 10.1093/cid/ciaa293, PMID: 32185385 PMC7744987

[6] Zhang N Wang R Li X Liu X Tang Z Liu Y. Are *Ureaplasma* spp. a cause of nongonococcal urethritis? A systematic review and meta-analysis. PLoS One. (2014) 9:e113771. doi: 10.1371/journal.pone.0113771, PMID: 25463970 PMC4252037

[7] Huang C Zhu HL Xu KR Wang SY Fan LQ Zhu WB. Mycoplasma and ureaplasma infection and male infertility: a systematic review and meta-analysis. Andrology. (2015) 3:809–16. doi: 10.1111/andr.12078, PMID: 26311339

[8] Lyss SB Kamb ML Peterman TA Moran JS Newman DR Bolan G. *Chlamydia trachomatis* among patients infected with and treated for *Neisseria gonorrhoeae* in sexually transmitted disease clinics in the United States. Ann Intern Med. (2003) 139:178–85. doi: 10.7326/0003-4819-139-3-200308050-00007, PMID: 12899585

[9] Dionne-Odom J Workowski K Perlowski C Taylor SN Mayer KH McNeil CJ. Coinfection with chlamydial and gonorrheal infection among US adults with early syphilis. Sex Transm Dis. (2022) 49:e87–9. doi: 10.1097/OLQ.0000000000001605, PMID: 35067599 PMC9283199

[10] Xu X Bradshaw CS Chow EPF Ong JJ Hocking JS Fairley CK. Modelling the multiple anatomical site transmission of *Mycoplasma genitalium* among men who have sex with men in Australia. Sci Rep. (2021) 11:11087. doi: 10.1038/s41598-021-90627-3, PMID: 34045569 PMC8160207

[11] Xu X Chow EPF Ong JJ Hoebe C Zou Z Hocking JS. *Chlamydia trachomatis* transmission between the oropharynx, urethra and anorectum in men who have sex with men: a mathematical model. BMC Med. (2020) 18:326. doi: 10.1186/s12916-020-01796-3, PMID: 33198750 PMC7670797

[12] Workowski KA Bachmann LH Chan PA Johnston CM Muzny CA Park I. Sexually transmitted infections treatment guidelines, 2021. MMWR Recomm Rep. (2021) 70:1–187. doi: 10.15585/mmwr.rr7004a1, PMID: 34292926 PMC8344968

[13] Taiwan AIDS Society (2020). Guidelines for diagnosis and treatment of HIV/AIDS. Available at: http://www.aids-care.org.tw/journal/treatment.php (accessed October 8, 2023).

[14] Jensen JS Cusini M Gomberg M Moi H Wilson J Unemo M. 2021 European guideline on the management of *Mycoplasma genitalium* infections. J Eur Acad Dermatol Venereol. (2022) 36:641–50. doi: 10.1111/jdv.17972, PMID: 35182080

[15] Sun HY Chiang C Huang SH Guo WJ Chuang YC Huang YC. Three-stage pooled plasma hepatitis C virus RNA testing for the identification of acute HCV infections in at-risk populations. Microbiol Spectr. (2022) 10:e0243721. doi: 10.1128/spectrum.02437-21, PMID: 35499354 PMC9241589

[16] Yassin R Hanna J El Bikai R Mohtar J El Chaar M. Comparison of two commercial multiplex PCR assays for the detection of sexually transmitted infections. J Infect Dev Ctries. (2022) 16:333–8. doi: 10.3855/jidc.15279, PMID: 35298429

[17] Kebbi-Beghdadi C Aeby S Baud D Greub G. Evaluation of a multiplex real-time PCR assay for detecting *Chlamydia trachomatis* in vaginal samples. Diagnostics (Basel). (2022) 12:1141. doi: 10.3390/diagnostics12051141, PMID: 35626297 PMC9139926

[18] Robinet S Parisot F. Accreditation of a multiplex real time PCR assay for detection and semi-quantitative determination of pathogens responsible of sexually-transmitted infections. Ann Biol Clin (Paris). (2018) 76:459–76. doi: 10.1684/abc.2018.1360, PMID: 30078782

[19] Spornraft-Ragaller P Dumke R. Prevalence and antibiotic resistance of rectal mollicutes in HIV-infected men who have sex with men at the University Hospital of Dresden, Germany. Infection. (2020) 48:259–65. doi: 10.1007/s15010-019-01386-3, PMID: 31993971 PMC7292812

[20] Kato Y Kawaguchi S Shigehara K Yaegashi H Nakashima K Nakagawa T. Prevalence of *N. gonorrhoeae*, *C. trachomatis*, *M. genitalium*, *M. Hominis* and *Ureaplasma* spp. in the anus and urine among Japanese HIV-infected men who have sex with men. J Infect Chemother. (2020) 26:403–6. doi: 10.1016/j.jiac.2019.12.007, PMID: 31882383

[21] Lee TF Lin KY Chang SY Huang YT Hsueh PR. Performance of two commercial multiplex polymerase chain reaction assays for the etiological diagnosis of sexually transmitted infections among men who have sex with men. J Microbiol Immunol Infect. (2023) 56:104–10. doi: 10.1016/j.jmii.2022.08.009, PMID: 36050217

[22] Cox C McKenna JP Watt AP Coyle PV. Ureaplasma parvum and *Mycoplasma genitalium* are found to be significantly associated with microscopy-confirmed urethritis in a routine genitourinary medicine setting. Int J STD AIDS. (2016) 27:861–7. doi: 10.1177/0956462415597620, PMID: 26378187

[23] Tao G Peterman TA Gift TL Nye MB. Syphilis testing among men who have had rectal gonorrhea and chlamydia tests, United States. J Epidemiol Glob Health. (2019) 9:153–7. doi: 10.2991/jegh.k.190620.001, PMID: 31529931 PMC7310823

[24] Rob F Jůzlová K Kružicová Z Vaňousová D Lásiková Š Sýkorová B. Prevalence of chlamydia trachomatis and *Neisseria gonorrhoeae* co-infections among patients with newly diagnosed syphilis: a single-Centre, cross-sectional study. Cent Eur J Public Health. (2019) 27:285–91. doi: 10.21101/cejph.a5142, PMID: 31951687

[25] World Health Organization (2021). Global progress report on HIV, viral hepatitis and sexually transmitted infections, 2021. Available at: http://who.int/publications/i/item/9789240027077 (accessed March 3, 2024).

[26] Unemo M Bradshaw CS Hocking JS de Vries HJC Francis SC Mabey D. Sexually transmitted infections: challenges ahead. Lancet Infect Dis. (2017) 17:e235–79. doi: 10.1016/S1473-3099(17)30310-9 28701272

[27] Wang H Zhang L Zhou Y Wang K Zhang X Wu J. The use of geosocial networking smartphone applications and the risk of sexually transmitted infections among men who have sex wit hmen: a systematic review and meta-analysis. BMC Public Health. (2018) 18:1178. doi: 10.1186/s12889-018-6092-3, PMID: 30326887 PMC6192100

[28] Chan PA Robinette A Montgomery M Almonte A Cu-Uvin S Lonks JR. Extragenital infections caused by *chlamydia trachomatis* and *Neisseria gonorrhoeae*: a review of the literature. Infect Dis Obstet Gynecol. (2016) 2016:5758387. doi: 10.1155/2016/5758387, PMID: 27366021 PMC4913006

[29] Chow EP Fairley CK. The role of saliva in gonorrhoea and chlamydia transmission to extragenital sites among men who have sex with men: new insights into transmission. J Int AIDS Soc. (2019) 22:e25354. doi: 10.1002/jia2.25354, PMID: 31468730 PMC6715946

[30] Zucker R Gaisa M Sigel K Singer I Adler A Turner D. Triple site sexually transmitted infection testing as a crucial component of surveillance for men who have sex with men: a prospective cohort study. Int J STD AIDS. (2022) 33:114–22. doi: 10.1177/09564624211047477, PMID: 34676780

[31] Tsai CS Chen PL Lee NY Tsai HP Huang SH Chen SY. Characteristics of rectal chlamydia among men who have sex with men in southern Taiwan, 2020-2022: an emerging threat of rectal lymphogranuloma venereum L2b. J Microbiol Immunol Infect. (2023) 56:408–15. doi: 10.1016/j.jmii.2023.01.007, PMID: 36682913

[32] Sultan B White JA Fish R Carrick G Brima N Copas A. The "3 in 1" study: pooling self-taken pharyngeal, urethral, and rectal samples into a single sample for analysis for detection of *Neisseria gonorrhoeae* and *Chlamydia trachomatis* in men who have sex with men. J Clin Microbiol. (2016) 54:650–6. doi: 10.1128/JCM.02460-15, PMID: 26719439 PMC4767962

[33] Chen KH Sun HY Chen CH Chuang YC Huang YS Liu WD. Higher serologic responses of early syphilis to single-dose benzathine penicillin G plus doxycycline versus single-dose benzathine penicillin G alone among people with HIV. Clin Infect Dis. (2023) 27:ciad 508. doi: 10.1093/cid/ciad508, PMID: 37633659

[34] Luetkemeyer AF Donnell D Dombrowski JC Cohen S Grabow C Brown C. (2023). Doxy PEP and antimicrobial resistance in *Neisseria gonorrhoeae*, commensal Neisseria and *Staphylococcus aureus*. Abstract of the Conference on Retroviruses and Opportunistic Infections.

[35] Kong FYS Kenyon C Unemo M. Important considerations regarding the widespread use of doxycycline chemoprophylaxis against sexually transmitted infections. J Antimicrob Chemother. (2023) 78:1561–8. doi: 10.1093/jac/dkad129, PMID: 37129293 PMC10577522

