# Supplementary material for: Sexually transmitted coinfections among at-risk HIV-positive MSM: implications for optimal preemptive treatment
Source: Front Med (Lausanne). 2024 Mar 15;11:1328589. doi: 10.3389/fmed.2024.1328589 (PMC10978595; doi:10.3389/fmed.2024.1328589)
Supplement: Supplementary file 3 [file Table_1.DOCX]

**Supplementary Materials**

Supplement to:

**Sexually transmitted coinfections among at-risk HIV-positive MSM: implications for optimal preemptive treatment**

Tzong-Yow Wu, Kuan-Yin Lin, Li-Hsin Su, Hsin-Yun Sun, Yu-Shan Huang, Wang-Da Liu, Wen-Chun Liu, Lan-Hsin Chang, Sui-Yuan Chang, Chien-Ching Hung

**Contents:**

Table S1.　　 Factors associated with *Chlamydia trachomatis* infection among the participants

Table S2.　　 Factors associated with *Neisseria gonorrhoeae* infection among the participants

Table S3.　　 Factors associated with *Mycoplasma genitalium* infection among the participants

Table S4.　　 Factors associated with *M. hominis* infection among the participants

Table S5.　　 Factors associated with *Ureaplasma urealyticum* infection among the participants

Table S6.　　 Factors associated with *U. parvum* infection among the participants

Figure S1. Proportions of concurrent bacterial sexually transmitted infections among the participants with and those without early syphilis

Figure S2. Additional benefits of different treatment regimens in managing concurrent bacterial sexually transmitted infections

**Table S1. Factors associated with *Chlamydia trachomatis* infection among the participants**

|  | **Univariable analysis** | | **Multivariable analysis^b^** | |
| --- | --- | --- | --- | --- |
|  | **OR (95% CI)** | ***P*** | **AOR (95% CI)** | ***P*** |
| Age, per 1-year increase | 0.99 (0.97-1.02) | 0.449 |  |  |
| HBsAg positivity | 0.98 (0.47-2.06) | 0.968 |  |  |
| HCV infection^a^ | 0.72 (0.44-1.18) | 0.198 |  |  |
| History of syphilis | 1.71 (0.89-3.29) | 0.106 |  |  |
| Early syphilis | 1.94 (1.25-3.00) | 0.003 | 1.66 (1.05-2.63) | 0.031 |
| Late syphilis | 0.62 (0.38-1.02) | 0.058 |  |  |
| Receiving cART at screening | 1.09 (0.30-3.95) | 0.901 |  |  |
| CD4 count at screening, per 10-cell/mm^3^ increase | 1.00 (0.99-1.01) | 0.780 |  |  |
| PVL at screening, per 1-log copies/mL increase | 1.02 (0.81-1.29) | 0.836 |  |  |
| Symptoms of STIs at screening | 1.00 (0.48-2.08) | 0.990 |  |  |
| Previous STIs within 1 year | 0.84 (0.56-1.25) | 0.386 |  |  |
| University or higher education | 1.41 (0.74-2.69) | 0.294 |  |  |
| Full-time employment | 0.97 (0.55-1.70) | 0.904 |  |  |
| Monthly income >1,600 USD | 1.49 (0.96-2.33) | 0.076 | 1.44 (0.92-2.26) | 0.110 |
| Substance use | 0.87 (0.56-1.36) | 0.543 |  |  |
| Anal-penile sex | 1.22 (0.70-2.13) | 0.488 |  |  |
| Oral-penile sex | 0.96 (0.59-1.58) | 0.883 |  |  |
| Oral-anal sex | 0.75 (0.47-1.19) | 0.227 |  |  |
| Partner known to have STI | 1.21 (0.78-1.88) | 0.401 |  |  |
| Number of sex partners >5 | 1.23 (0.67-2.24) | 0.500 |  |  |
| Inconsistent condom use | 1.00 (0.62-1.62) | 0.997 |  |  |
| Use of mobile dating application | 1.39 (0.88-2.20) | 0.163 |  |  |
| Chemsex | 0.67 (0.36-1.25) | 0.206 |  |  |

**Abbreviations:** AOR, adjusted odds ratio; cART, combination antiretroviral therapy; CI, confidence interval; HBsAg, hepatitis B surface antigen; HCV hepatitis C virus; PVL, plasma HIV RNA load; STI, sexually transmitted infection; USD, United States dollars.

^a^Participants testing positive for anti-HCV IgG and/or HCV RNA in the past 12 months.

^b^The ORs are the estimates of the effect of covariates on *C. trachomatis* infection, adjusted for early syphilis and monthly income using a logistic regression model. Late syphilis was excluded from the multivariable analysis due to collinearity with early syphilis.

**Table S2. Factors associated with *Neisseria gonorrhoeae* infection among the participants**

|  | **Univariable analysis** | | **Multivariable analysis^b^** | |
| --- | --- | --- | --- | --- |
|  | **OR (95% CI)** | ***P*** | **AOR (95% CI)** | ***P*** |
| Age, per 1-year increase | 0.95 (0.92-0.98) | <0.001 | 0.94 (0.91-0.98) | 0.001 |
| HBsAg positivity | 0.60 (0.25-1.46) | 0.257 |  |  |
| HCV infection^a^ | 1.02 (0.63-1.67) | 0.930 |  |  |
| History of syphilis | 1.25 (0.66-2.37) | 0.486 |  |  |
| Early syphilis | 1.05 (0.68-1.63) | 0.812 |  |  |
| Late syphilis | 1.07 (0.66-1.72) | 0.793 |  |  |
| Receiving cART at screening | 1.50 (0.33-6.79) | 0.601 |  |  |
| CD4 count at screening, per 10-cell/mm^3^ increase | 1.01 (1.00-1.02) | 0.026 | 1.01 (1.00-1.02) | 0.042 |
| PVL at screening, per 1-log copies/mL increase | 1.06 (0.84-1.34) | 0.602 |  |  |
| Symptoms of STIs at screening | 4.36 (2.31-8.24) | <0.001 | 4.00 (1.96-8.21) | <0.001 |
| Previous STIs within 1 year | 1.04 (0.68-1.59) | 0.868 |  |  |
| University or higher education | 0.58 (0.33-1.03) | 0.063 | 0.59 (0.31-1.11) | 0.099 |
| Full-time employment | 1.21 (0.65-2.22) | 0.547 |  |  |
| Monthly income >1,600 USD | 1.24 (0.78-1.97) | 0.358 |  |  |
| Substance use | 1.46 (0.93-2.30) | 0.099 | 1.08 (0.65-1.77) | 0.776 |
| Anal-penile sex | 1.55 (0.85-2.84) | 0.154 |  |  |
| Oral-penile sex | 1.75 (1.00-3.06) | 0.051 | 1.53 (0.84-2.78) | 0.164 |
| Oral-anal sex | 1.32 (0.84-2.09) | 0.230 |  |  |
| Partner known to have STI | 1.39 (0.88-2.20) | 0.152 |  |  |
| Number of sex partners >5 | 2.65 (1.51-4.66) | 0.001 | 2.52 (1.34-4.71) | 0.004 |
| Inconsistent condom use | 1.42 (0.84-2.39) | 0.188 |  |  |
| Use of mobile dating application | 1.25 (0.79-2.00) | 0.344 |  |  |
| Chemsex | 1.49 (0.86-2.58) | 0.159 |  |  |

**Abbreviations:** AOR, adjusted odds ratio; cART, combination antiretroviral therapy; CI, confidence interval; HBsAg, hepatitis B surface antigen; HCV hepatitis C virus; PVL, plasma HIV RNA load; STI, sexually transmitted infection; USD, United States dollars.

^a^Participants testing positive for anti-HCV IgG and/or HCV RNA in the past 12 months.

^b^The ORs are the estimates of the effect of covariates on *N. gonorrhoeae* infection, adjusted for age, CD4 count, symptoms of STIs, education, substance use, oral-penile sex, and number of sex partners using a logistic regression model.

**Table S3. Factors associated with *Mycoplasma genitalium* infection among the participants**

|  | **Univariable analysis** | | **Multivariable analysis^b^** | |
| --- | --- | --- | --- | --- |
|  | **OR (95% CI)** | ***P*** | **AOR (95% CI)** | ***P*** |
| Age, per 1-year increase | 0.97 (0.94-1.01) | 0.153 |  |  |
| HBsAg positivity | 1.97 (0.87-4.49) | 0.105 |  |  |
| HCV infection^a^ | 1.07 (0.570-2.01) | 0.829 |  |  |
| History of syphilis | 1.03 (0.47-2.28) | 0.935 |  |  |
| Early syphilis | 1.30 (0.73-2.31) | 0.368 |  |  |
| Late syphilis | 0.72 (0.37-1.41) | 0.338 |  |  |
| Receiving cART at screening | 0.28 (0.09-0.93) | 0.038 | 1.17 (0.15-9.06) | 0.880 |
| CD4 count at screening, per 10-cell/mm^3^ increase | 1.00 (0.99-1.01) | 0.977 |  |  |
| PVL at screening, per 1-log copies/mL increase | 1.37 (1.09-1.72) | 0.006 | 1.40 (0.95-2.06) | 0.087 |
| Symptoms of STIs at screening | 0.38 (0.09-1.60) | 0.187 |  |  |
| Previous STIs within 1 year | 1.19 (0.68-2.08) | 0.546 |  |  |
| University or higher education | 0.74 (0.35-1.56) | 0.434 |  |  |
| Full-time employment | 0.88 (0.42-1.84) | 0.733 |  |  |
| Monthly income >1,600 USD | 0.46 (0.23-0.92) | 0.027 | 0.50 (0.24-1.04) | 0.063 |
| Substance use | 1.34 (0.75-2.40) | 0.325 |  |  |
| Anal-penile sex | 1.21(0.57-2.59) | 0.618 |  |  |
| Oral-penile sex | 0.82 (0.43-1.55) | 0.537 |  |  |
| Oral-anal sex | 1.30 (0.72-2.34) | 0.383 |  |  |
| Partner known to have STI | 0.55 (0.30-1.01) | 0.055 | 0.58 (0.31-1.09) | 0.088 |
| Number of sex partners >5 | 0.48 (0.17-1.37) | 0.171 |  |  |
| Inconsistent condom use | 1.00 (0.53-1.89) | 0.998 |  |  |
| Mobile dating application | 1.18 (0.64-2.15) | 0.599 |  |  |
| Chemsex | 1.45 (0.72-2.91) | 0.293 |  |  |

**Abbreviations:** AOR, adjusted odds ratio; cART, combination antiretroviral therapy; CI, confidence interval; HBsAg, hepatitis B surface antigen; HCV hepatitis C virus; PVL, plasma HIV RNA load; STI, sexually transmitted infection; USD, United States dollars.

^a^Participants testing positive for anti-HCV IgG and/or HCV RNA in the past 12 months.

^b^The ORs are the estimates of the effect of covariates on *M. genitalium* infection, adjusted for receiving cART, PVL, monthly income, and partner infected with STI using a logistic regression model.

**Table S4. Factors associated with *M. hominis* infection among the participants**

|  | **Univariable analysis** | | **Multivariable analysis^b^** | |
| --- | --- | --- | --- | --- |
|  | **OR (95% CI)** | ***P*** | **AOR (95% CI)** | ***P*** |
| Age, per 1-year increase | 0.99 (0.96-1.02) | 0.464 |  |  |
| HBsAg positivity | 0.58 (0.22-1.51) | 0.264 |  |  |
| HCV infection^a^ | 1.74 (1.07-2.81) | 0.024 | 1.48 (0.83-2.64) | 0.188 |
| History of syphilis | 2.35 (1.04-5.28) | 0.039 |  |  |
| Early syphilis | 1.77 (1.10-2.87) | 0.019 | 1.65 (0.94-2.89) | 0.084 |
| Late syphilis | 0.77 (0.45-1.31) | 0.331 |  |  |
| Receiving cART at screening | 0.37 (0.12-1.13) | 0.082 | 1.44 (0.18-11.34) | 0.731 |
| CD4 count at screening, per 10-cell/mm^3^ increase | 0.99 (0.98-1.00) | 0.036 | 0.99 (0.97-1.00) | 0.010 |
| PVL at screening, per 1-log copies/mL increase | 1.34 (1.08-1.65) | 0.007 | 1.25 (0.85-1.84) | 0.265 |
| Symptoms of STIs at screening | 2.40 (1.22-4.72) | 0.011 | 2.07 (0.92-4.65) | 0.077 |
| Previous STIs within 1 year | 1.08 (0.69-1.69) | 0.737 |  |  |
| University or higher education | 0.57 (0.32-1.04) | 0.069 | 0.58 (0.30-1.15) | 0.118 |
| Full-time employment | 1.00 (0.53-1.89) | 0.993 |  |  |
| Monthly income >1,600 USD | 0.74 (0.44-1.24) | 0.249 |  |  |
| Substance use | 1.53 (0.94-2.49) | 0.089 | 0.93 (0.48-1.81) | 0.831 |
| Anal-penile sex | 1.99 (0.90-4.03) | 0.056 | 1.77 (0.80-3.93) | 0.161 |
| Oral-penile sex | 1.07 (0.61-1.86) | 0.817 |  |  |
| Oral-anal sex | 1.17 (0.71-1.90) | 0.541 |  |  |
| Partner known to have STI | 1.15 (0.70-1.87) | 0.587 |  |  |
| Number of sex partners >5 | 1.36 (0.71-2.59) | 0.356 |  |  |
| Inconsistent condom use | 1.33 (0.77-2.32) | 0.305 |  |  |
| Use of mobile dating application | 0.76 (0.47-1.23) | 0.263 |  |  |
| Chemsex | 2.39 (1.37-4.17) | 0.002 | 2.37 (1.14-4.95) | 0.022 |

**Abbreviations:** AOR, adjusted odds ratio; cART, combination antiretroviral therapy; CI, confidence interval; HBsAg, hepatitis B surface antigen; HCV hepatitis C virus; PVL, plasma HIV RNA load; STI, sexually transmitted infection; USD, United States dollars.

^a^Participants testing positive for anti-HCV IgG and/or HCV RNA in the past 12 months.

^b^The ORs are the estimates of the effect of covariates on *M. hominis* infection, adjusted for HCV infection, early syphilis, receiving cART, CD4 count, PVL, symptoms of STIs, education, substance use, anal-penile sex, and chemsex using a logistic regression model.

**Table S5. Factors associated with *Ureaplasma urealyticum* infection among the participants**

|  | **Univariable analysis** | | **Multivariable analysis^b^** | |
| --- | --- | --- | --- | --- |
|  | **OR (95% CI)** | ***P*** | **AOR (95% CI)** | ***P*** |
| Age, per 1-year increase | 0.99 (0.97-1.01) | 0.235 |  |  |
| HBsAg positivity | 1.66 (0.89-3.09) | 0.108 |  |  |
| HCV infection^a^ | 1.48 (0.99-2.20) | 0.056 |  |  |
| History of syphilis | 2.22 (1.26-3.93) | 0.006 |  |  |
| Early syphilis | 1.85 (1.28-2.68) | 0.001 | 2.00 (1.31-3.06) | 0.001 |
| Late syphilis | 0.74 (0.49-1.11) | 0.146 |  |  |
| Receiving cART at screening | 1.44 (0.44-4.64) | 0.546 |  |  |
| CD4 count at screening, per 10-cell/mm^3^ increase | 1.00 (0.99-1.00) | 0.333 |  |  |
| PVL at screening, per 1-log copies/mL increase | 0.90 (0.72-1.12) | 0.360 |  |  |
| Symptoms of STIs at screening | 1.00 (0.53-1.91) | 0.988 |  |  |
| Previous STIs within 1 year | 1.68 (1.17-2.41) | 0.005 | 1.19 (0.77-1.85) | 0.433 |
| University or higher education | 0.72 (0.43-1.19) | 0.201 |  |  |
| Full-time employment | 1.26 (0.76-2.08) | 0.377 |  |  |
| Monthly income >1,600 USD | 1.19 (0.81-1.76) | 0.378 |  |  |
| Substance use | 1.42 (0.97-2.09) | 0.069 | 1.15 (0.75-1.75) | 0.522 |
| Anal-penile sex | 1.12 (0.70-1.79) | 0.633 |  |  |
| Oral-penile sex | 1.27 (0.82-1.96) | 0.288 |  |  |
| Oral-anal sex | 1.18 (0.80-1.75) | 0.392 |  |  |
| Partner known to have STI | 1.03 (0.70-1.52) | 0.864 |  |  |
| Number of sex partners >5 | 2.34 (1.38-3.98) | 0.002 | 2.15 (1.23-3.75) | 0.007 |
| Inconsistent condom use | 1.66 (1.07-2.56) | 0.023 | 1.37 (0.86-2.19) | 0.187 |
| Use of mobile dating application | 1.52 (1.02-2.25) | 0.038 | 1.35 (0.88-2.06) | 0.166 |
| Chemsex | 1.47 (0.91-2.39) | 0.119 |  |  |

**Abbreviations:** AOR, adjusted odds ratio; cART, combination antiretroviral therapy; CI, confidence interval; HBsAg, hepatitis B surface antigen; HCV hepatitis C virus; PVL, plasma HIV RNA load; STI, sexually transmitted infection; USD, United States dollars.

^a^Participants testing positive for anti-HCV IgG and/or HCV RNA in the past 12 months.

^b^The ORs are the estimates of the effect of covariates on *U. urealyticum* infection, adjusted for early syphilis, previous STIs within 1 year, substance use, number of sex partners, inconsistent condom use, and mobile dating application using a logistic regression model.

**Table S6. Factors associated with *U. parvum* infection among the participants**

|  | **Univariable analysis** | | **Multivariable analysis^c^** | |
| --- | --- | --- | --- | --- |
|  | **OR (95% CI)** | ***P*** | **AOR (95% CI)** | ***P*** |
| Age, per 1-year increase | 1.05 (0.96-1.16) | 0.245 |  |  |
| HBsAg positivity | 5.60 (1.00-31.45) | 0.051 | 6.78 (1.07-42.97) | 0.042 |
| HCV infection^a^ | 3.02 (0.60-15.16) | 0.179 |  |  |
| History of syphilis | 0.84 (0.10-7.28) | 0.873 |  |  |
| Early syphilis | 1.37 (0.25-7.57) | 0.715 |  |  |
| Late syphilis | 0.56 (0.06-4.80) | 0.593 |  |  |
| Receiving cART at screening | -^b^ | -^b^ |  |  |
| CD4 count at screening, per 10-cell/mm^3^ increase | 1.00 (0.97-1.03) | 0.828 |  |  |
| PVL at screening, per 1-log copies/mL increase | 0.99 (0.38-2.56) | 0.978 |  |  |
| Symptoms of STIs at screening | 2.26 (0.26-19.79) | 0.461 |  |  |
| Previous STIs within 1 year | 1.59 (0.29-8.74) | 0.596 |  |  |
| University or higher education | 0.27 (0.04-1.64) | 0.156 |  |  |
| Full-time employment | -^b^ | -^b^ |  |  |
| Monthly income >1,600 USD | 7.52 (0.83-67.87) | 0.072 | 7.11 (0.78-64.84) | 0.082 |
| Substance use | 0.90 (0.15-5.43) | 0.907 |  |  |
| Anal-penile sex | -^b^ | -^b^ |  |  |
| Oral-penile sex | 1.44 (0.16-12.99) | 0.747 |  |  |
| Oral-anal sex | 0.38 (0.04-3.43) | 0.388 |  |  |
| Partner known to have STI | 1.52 (0.25-9.19) | 0.648 |  |  |
| Number of sex partners >5 | -^b^ | -^b^ |  |  |
| Inconsistent condom use | 1.67 (0.19-15.12) | 0.646 |  |  |
| Use of mobile dating application | 0.45 (0.07-2.74) | 0.388 |  |  |
| Chemsex | 3.13 (0.51-19.05) | 0.215 |  |  |

**Abbreviations:** AOR, adjusted odds ratio; cART, combination antiretroviral therapy; CI, confidence interval; HBsAg, hepatitis B surface antigen; HCV hepatitis C virus; PVL, plasma HIV RNA load; STI, sexually transmitted infection; USD, United States dollars.

^a^Participants testing positive for anti-HCV IgG and/or HCV RNA in the past 12 months.

^b^All participants testing positive for *U. parvum* had been receiving cART at screening, as well as had full-time employment, anal-penile sex, and ≤5 sex partners.

^c^The ORs are the estimates of the effect of covariates on *U. parvum* infection, adjusted for HBsAg positivity and monthly income using a logistic regression model.

**Figure S1. Proportions of concurrent bacterial sexually transmitted infections among the participants with and those without early syphilis**

**
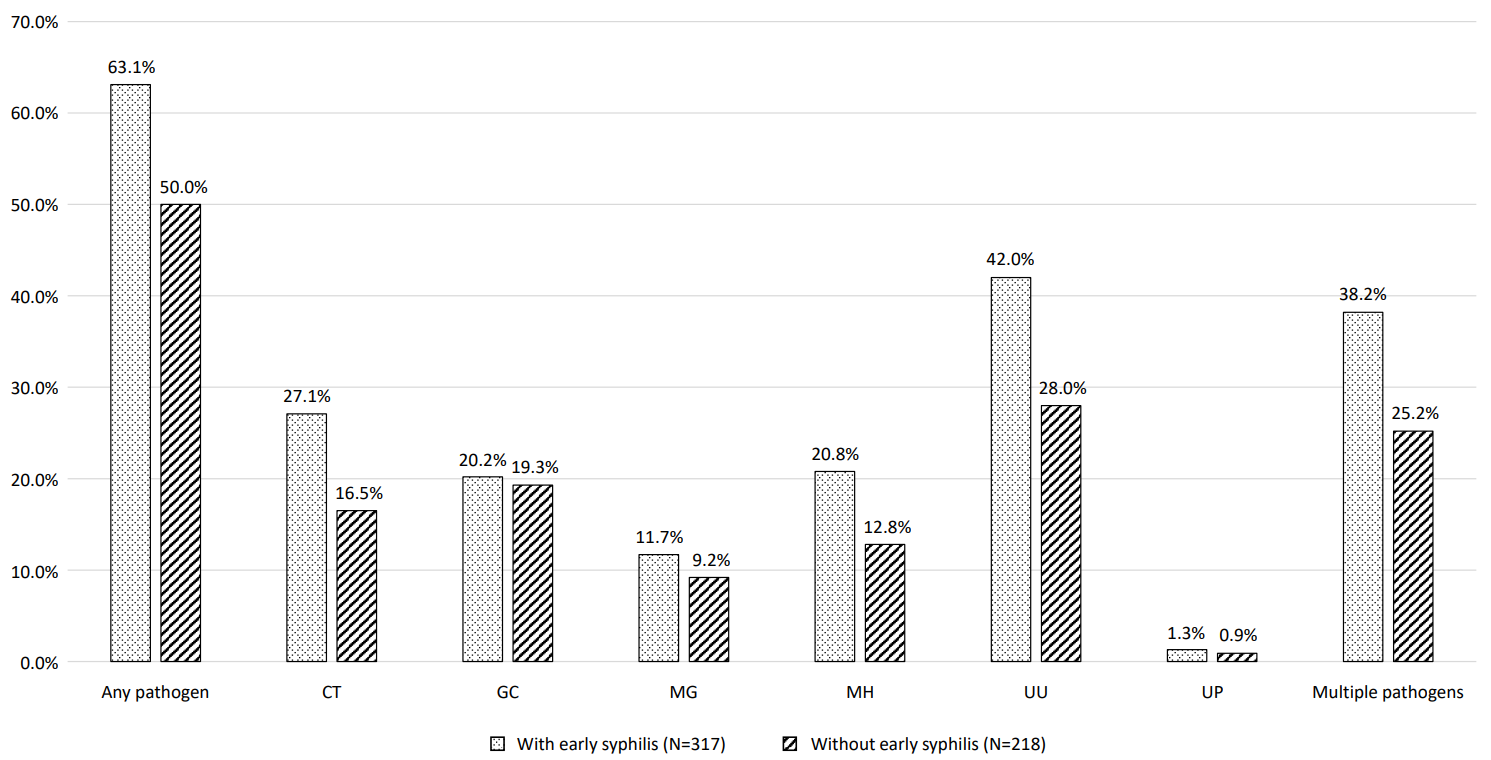
**

**Abbreviations:** CT, *Chlamydia trachomatis*; GC, *Neisseria gonorrhoeae*; MG, *Mycoplasma genitalium*; MH, *Mycoplasma hominis*; STI, sexually transmitted infection; UU, *Ureaplasma urealyticum*; UP, *Ureaplasma parvum*.

**Figure S2. Additional benefits of different treatment regimens in managing concurrent bacterial sexually transmitted infections. (A) A single dose of intramuscular benzathine penicillin G (2.4 million units), (B) A single dose of intramuscular benzathine penicillin G (2.4 million units) plus a 7-day course of oral doxycycline (100 mg twice daily), (C) A single dose of intramuscular benzathine penicillin G (2.4 million units) plus a single dose of oral azithromycin (1 g), (D) A single dose of intramuscular ceftriaxone (500 mg), (E) A single dose of intramuscular ceftriaxone (500 mg) plus a 7-day course of oral doxycycline (100 mg twice daily), (F) A single dose of intramuscular ceftriaxone (500 mg) plus a single dose of oral azithromycin (1 g), (G) A 14-day course of oral doxycycline (100 mg twice daily).**


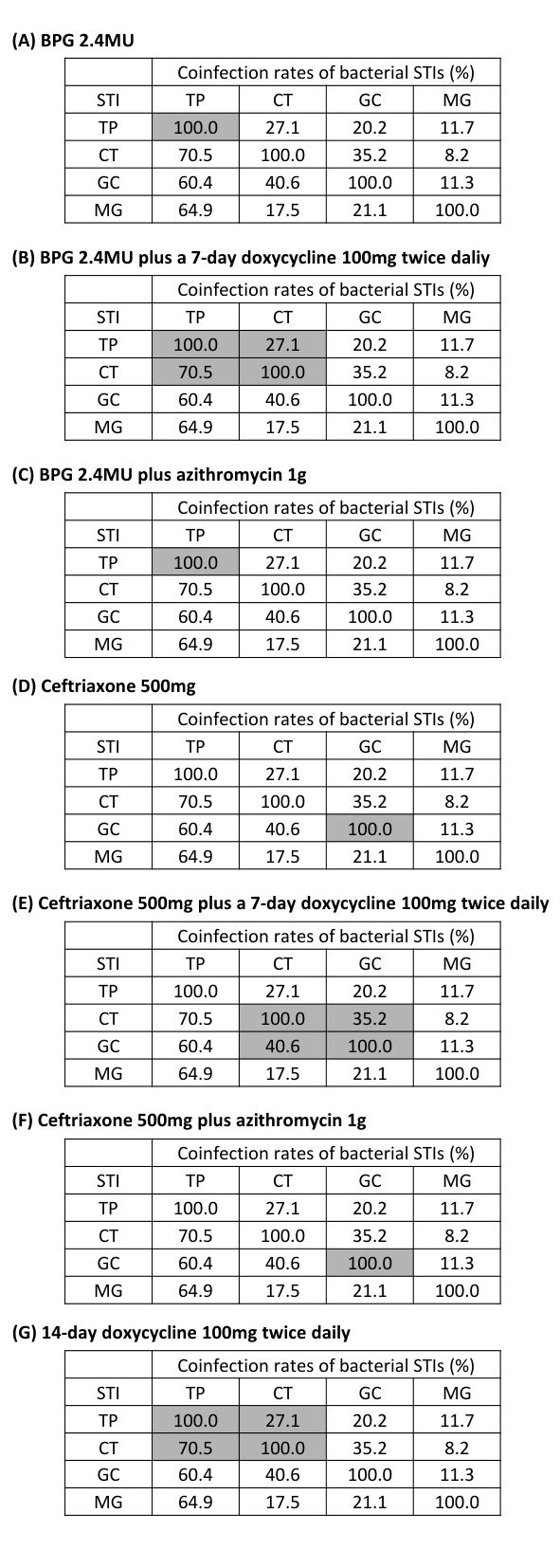


**Abbreviations:** STI, sexually transmitted infection; BPG, benzathine penicillin G; TP, early syphilis; CT, *Chlamydia trachomatis*; GC, *Neisseria gonorrhoeae*; MG, *Mycoplasma genitalium*; MH, *Mycoplasma hominis*; UU, *Ureaplasma urealyticum*; UP, *Ureaplasma parvum*.

*Dark gray backgrounds indicate that treatment regimens are recommended for the coinfections according to the US guidelines for STIs (1). For early syphilis, the recommended treatment is a single dose of intramuscular benzathine penicillin G (BPG) at 2.4 million units (MU), with a 14-day course of oral doxycycline (100 mg twice daily) considered as an alternative regimen. For gonorrhea, a single dose of intramuscular ceftriaxone (500 mg) is suggested. Chlamydia treatment involves a 7-day course of oral doxycycline for chlamydia, while *M. genitalium* infection requires sequential doxycycline followed by either azithromycin or moxifloxacin. Azithromycin is suggested as an alternative regimen for early syphilis and chlamydia; however, it is not recommended as a first-line therapy because of increasing resistance and reduced clinical efficacy. Although *M. hominis* and *Ureaplasma* spp. can also cause urethritis, routine testing and treatment for these pathogens are generally not recommended (2).

**Reference:**

1. Workowski KA, Bachmann LH, Chan PA, Johnston CM, Muzny CA, Park I, et al. Sexually transmitted infections treatment guidelines, 2021. *MMWR Recomm Rep*. (2021) 70:1-187. doi: 10.15585/mmwr.rr7004a1

2. Horner P, Donders G, Cusini M, Gomberg M, Jensen JS, Unemo M. Should we be testing for urogenital *Mycoplasma hominis*, *Ureaplasma parvum* and *Ureaplasma urealyticum* in men and women? —A position statement from the European STI Guidelines Editorial Board. *J Eur Acad Dermatol Venereol.* (2018) 32:1845-1851. doi: 10.1111/jdv.15146
